# Direct experimental determination of the topological winding number of skyrmions in Cu_2_OSeO_3_

**DOI:** 10.1038/ncomms14619

**Published:** 2017-02-24

**Authors:** S. L. Zhang, G. van der Laan, T. Hesjedal

**Affiliations:** 1 Clarendon Laboratory, Department of Physics, University of Oxford, Parks Road, Oxford OX1 3PU, UK; 2 Magnetic Spectroscopy Group, Diamond Light Source, Didcot OX11 0DE, UK

## Abstract

The mathematical concept of topology has brought about significant advantages that allow for a fundamental understanding of the underlying physics of a system. In magnetism, the topology of spin order manifests itself in the topological winding number which plays a pivotal role for the determination of the emergent properties of a system. However, the direct experimental determination of the topological winding number of a magnetically ordered system remains elusive. Here, we present a direct relationship between the topological winding number of the spin texture and the polarized resonant X-ray scattering process. This relationship provides a one-to-one correspondence between the measured scattering signal and the winding number. We demonstrate that the exact topological quantities of the skyrmion material Cu_2_OSeO_3_ can be directly experimentally determined this way. This technique has the potential to be applicable to a wide range of materials, allowing for a direct determination of their topological properties.

## Abstract (web summary)

Experimental demonstrations of topologically nontrivial states in magnetic films currently rely on indirect comparisons of theoretical models with microscopic images. Here the authors show that resonant X-ray scattering provides direct information on the topology of magnetic textures.

In a many-body system, a local order parameter can be assigned to individual entities, and by considering interactions among them, emergent phases and novel physical properties may evolve. The possible values of the order parameters constitute the order parameter space, which can be described in the framework of topology^1^,^2^. In magnetism, the spins are the elementary entities, and the order parameter is the magnetization vector **m**. Its magnitude can be taken as a constant, that is, its three components satisfy 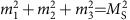, where *M*_S_ is the saturation magnetization. Therefore, the order parameter space is the surface of a three-dimensional unit sphere, which is described by the homotopy group *π*_2_(*S*^2^) for a two-dimensional physical space (*x*, *y*) (ref. 2). Different homotopy classes with distinct topological properties can be quantified based on the winding number *N*, which is an integer that counts the number of times the physical space fully covers the order parameter space^3^. It is defined as


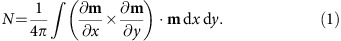


Recently, it has been demonstrated that non-centrosymmetric helimagnetic materials carry *N*=1 magnetic skyrmions^4^,^5^,^6^, which leads to emergent phenomena, including novel magneto-electrical transport effects (that is, the topological Hall effect^7^,^8^,^9^, skyrmion motion induced by ultralow current densities^10^,^11^,^12^,^13^, and emergent electromagnetic fields^14^,^15^,^16^), as well as new spin dynamic properties^17^,^18^,^19^. Utilizing this nontrivial topological order, advanced spintronics applications have been devised^20^,^21^,^22^,^23^,^24^. More recently, several candidates with *N*=2 have also been discovered^25^,^26^, suggesting that other elements from the *π*_2_(*S*^2^) group may exist in nature as well.

While the significance of the topological properties of ordered systems is being recognized more and more, the experimental determination of the winding number for spin-ordered media remains challenging. Commonly, the winding number is determined by comparing a microscopic image of the magnetization state with theoretical model calculations, making it a rather indirect process that has no unique answer^21^,^22^,^23^,^24^,^27^,^28^,^29^. Most importantly, the established magnetic imaging techniques only give a partial picture of the local magnetization vector, as they are both limited in three-dimensional sensitivity and lateral resolution. For example, Lorentz transmission electron microscopy (LTEM) has been the most common technique which is used to infer the topological winding number from a magnetization map^27^. In most of the LTEM experiments, the magnetization configuration is obtained via an indirect transport-of-intensity equation simulation process, and, most importantly, the information of the out-of-plane spin component is missing^27^. Consequently, LTEM is not a direct experimental method^30^ to determine the topological winding number (see Supplementary Note 3 for a detailed discussion). On the other hand, the presence of skyrmions leads to measurable signals in electric transport, that is, the topological Hall effect^9^. Nevertheless, other non-collinear magnetic structures, which are not related to skyrmions, can also give rise to a measurable topological Hall effect^7^,^31^, rendering transport measurements less ideal for the unambiguous determination of topological properties.

Here, we show that the winding number *N* can be unambiguously identified by utilizing the sensitivity of the light polarization to the magnetic order at resonant elastic X-ray scattering (REXS) condition, referred as polarization-dependent REXS.

## Results

### Representation of a skyrmion with winding number *N*

A general magnetic skyrmion structure can be obtained by mapping the order parameter space to the physical space, described in the two-dimensional polar coordinates *ρ* (radial coordinate) and Ψ (azimuthal angle), in the following way^4^,^32^:


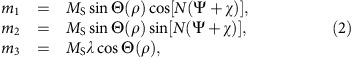


where the boundary conditions are defined such that the magnetization points up in the centre of the two-dimensional physical space and down at the boundary. The function Θ(*ρ*) describes the radial profile of the out-of-plane component of the magnetization, starting from the centre and extending to the boundary; *χ* is the helicity, defined in the range of (−*π*, *π*]; and *λ* takes the values of ±1, describing the polarity of the skyrmion^4^. For example, an *N*=1 skyrmion appears as a vortex-like texture. If the core magnetization points up (that is, *λ*=1), a *χ*=−*π*/2 skyrmion has a clockwise rotation sense when viewing from the top. The entire texture thus carries negative chirality, defined by *C*=sgn(*λχ*). Analogously, a *χ*=*π*/2 skyrmion carries positive chirality. On the other hand, for *χ*=0 and *χ*=*π* skyrmions, so-called Néel-type skyrmions, there is no chirality present^33^.

**Figure 1:**
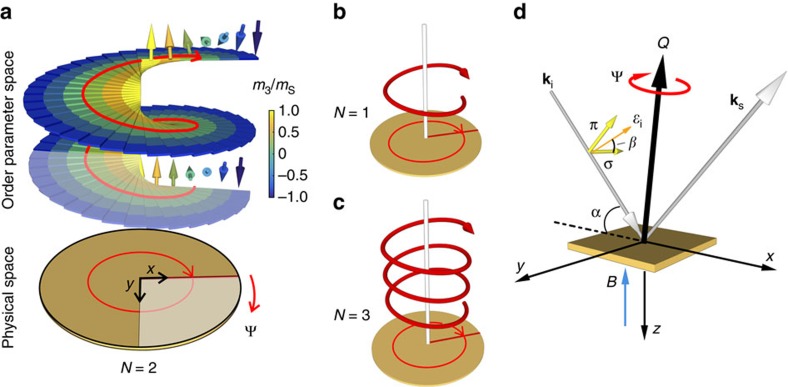
Concept of winding number and experimental setup. (**a**) For classical spins in two-dimensional space, the spin configuration that carries a winding number of *N*, as described by equation (1), can be equivalently constructed by mapping the two-dimensional physical space using one-dimensional helices. In order parameter space, the one-dimensional helices are rotated azimuthally by *N*Ψ from the base position (see, for example, bottom spin helix for Ψ=0°), and projected onto physical space at the azimuthal position Ψ. The helices are stacked up in order parameter space for illustrative purposes following the red helical guideline. The example shows the situation for *N*=2 where the order parameter space maps the physical space twice. The shaded quarter-circle in physical space (below) corresponds to a covered half-circle in order parameter space (above). Simplified plots for the *N*=1 and *N*=3 cases are shown in **b**,**c**, respectively. (**d**) When Ψ covers the range from 0° to 360°, the scattered intensity will exhibit a periodicity that only depends on *N*. Both circularly or linearly polarized incident light is used, allowing for two measurement strategies: CD plots and PAMs. In both cases, the diffraction condition is met for the wavevector **Q**, which contains the topological motif's modulation wavevector for different azimuthal angles Ψ. The polarization angle *β* of the incident light polarization vector 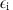 is defined with respect to *σ*-polarization (*β*=0°; *β*=90° corresponds to *π*-polarization).

Another, more illustrative way to interpret equation (2), is to construct an *N*-skyrmion texture by assigning a one-dimensional helix spin profile to a radial chain in physical space, and by repeating the process for all azimuthal angles Ψ, in the range from 0° to 360°, thereby mapping out the entire two-dimensional physical space. This concept is illustrated in Fig. 1a–c. Starting from the line for Ψ=0° that is parallel to *x* axis, a standard harmonic helix structure is assigned. Subsequent Ψ angles get a helix assigned that is rotated by *N*Ψ from Ψ=0°. As as result, when the two-dimensional physical space is fully sampled, the order parameter space will have been mapped out *N* times. The exact structure of such harmonic helices does not affect the topological properties of the system, nor our measurement principle, as will be shown below. Using this one-dimensional helix approximation, an analytical solution for the polarization-dependent REXS process can be obtained that is explicit in *N*.

### Topology determination principle

The measurement geometry for determining *N* is illustrated in Fig. 1d. The incident and scattered X-ray wavevectors are denoted as **k**_i_ and **k**_s_, with the incident angle *α*, which satisfies the diffraction condition **Q**=**k**_s_−**k**_i_. The incident X-rays can be linearly polarized with the polarization angle *β*. We define *β*=0° corresponding to *σ*-polarization, while *β*=90° corresponds to *π*-polarization. Alternatively, the light can be circularly polarized. Here, we define the circular dichroism (CD) signal as the difference of the scattering cross-sections for left-circularly and right-circularly polarized incident light (at the same diffraction condition).

We demonstrate our new experimental principle for the determination of *N* on the magnetic skyrmion system Cu_2_OSeO_3_. This material carries an incommensurate, hexagonal lattice with an *N*≠0 topological entity motif^34^,^35^,^36^. The modulation wavevector is ∼0.0158, r.l.u., and the motif's periodic lattice lies in the *x*–*y*-plane when the required magnetic field is along the *z* direction^37^ (see Fig. 1). Using the one-dimensional helix approximation construction, an analytical form of the resonant magnetic diffraction cross-section *I* and the CD cross-section *I*_CD_ can be derived as a function of *N* (see Methods for the derivation)


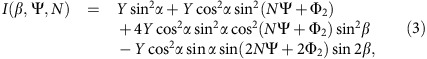


and


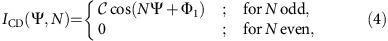


where 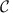 and *Y* are constants. The arbitrary phase parameters Φ_1_ and Φ_2_ can be chosen to adapt to other spin configurations with the same winding number, however, which deviate from the ‘standard' configuration as constructed in Fig. 1a. These two relationships can be interpreted in the following way, which form the core of our experimental method for the determination of the winding number: In case of an odd winding number, *N* equals to the periodicity of the CD signal, while Ψ covers the full range from 0° to 360°. *N* is also equal to half the number of peaks in the polarization-azimuthal map (PAM; see below). For an even winding number, no CD signal is observed. The case of non-integer winding numbers is discussed in Supplementary Note 2.

### Numerical results

**Figure 2:**
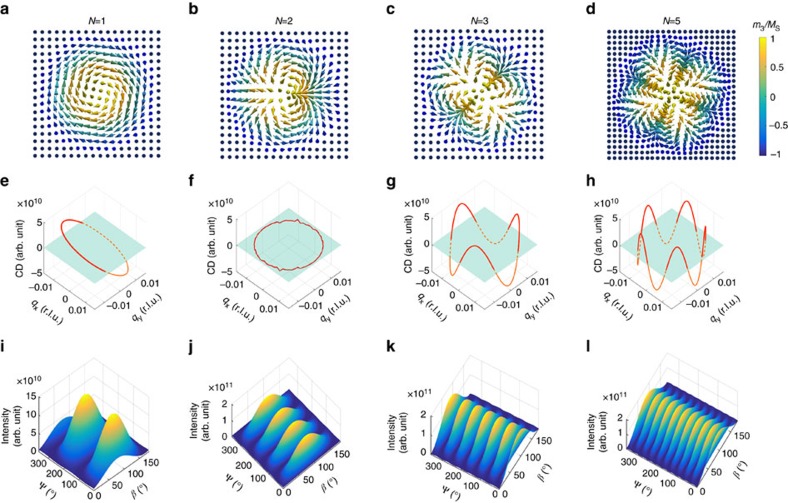
Numerical determination of the topological winding number. (**a**–**d**) Spin configurations with topological winding numbers of 1, 2, 3 and 5, respectively. These spin textures are the motifs that generate the two-dimensional, periodically ordered lattices which can be measured by diffraction techniques. (**e**–**h**) CD cross-section as a function of Ψ for topologically ordered systems. Here, Ψ is represented by a loop (shown in red) in reciprocal space (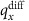, 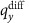), where 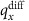
=*q* cos Ψ, 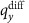
=*q* sin Ψ, and *q*=0.0158 r.l.u. is the absolute value of the skyrmion lattice modulation wavevector for Cu_2_OSeO_3_. The condition for vanishing CD (CD=0) is indicated by the light blue plane. The CD signal is symmetric about this plane for integer winding numbers. The periodicity of the CD modulation equals to *N*. Note that for even *N* the CD signal is zero. (**i**–**l**) PAMs. The calculations are performed by rotating *β* from 0° to 180° at each Ψ, and by mapping out Ψ from 0° to 360°. The total number of humps is equivalent to 2*N*. Note that for integer winding numbers the humps are of equal height.

Figure 2e–h shows the numerical calculation results for the CD cross-section for different topological spin motifs. Equation (4) can be represented by the CD amplitude as a function of a closed-loop in reciprocal space. Each reciprocal space point (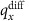, 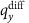) on the loop (shown in red) corresponds to one azimuthal angle at which the diffraction condition for the modulation wavevector for Ψ is met. Therefore, according to equation (4), the CD intensity varies as a function of Ψ, with a periodicity that is equal to *N* if the spin winding is an odd number. For even winding numbers, such as *N*=2, there is no CD. This can be understood by treating a *N*=2 skyrmion as two *N*=1 skyrmions pulled together^2^ (see Fig. 2b). These two *N*=1 skyrmions have opposite chirality, thus there is no global chirality of this spin configuration. As CD is sensitive to chiral structures^38^, the total CD for this state is zero. This also applies for other even winding number systems.

On the other hand, by varying the linear polarization *β* of the incident light from 0° to 180°, one can measure the polarization-dependent scattering intensity at each Ψ. By covering Ψ in the range from 0° to 360°, a PAM is obtained. The PAM plot in Fig. 2i–l shows hump-like, two-dimensional peaks of equal height. The peaks appear around *β*≈90°, and modulate along Ψ. The periodicity of the PAM signal is twice that of the winding number. This PAM feature is consistent with the analytical solution described by equation (3).

If both the CD and PAM data can be fitted by the two equations (3) and (4) simultaneously, the winding number can be unambiguously determined. If the detailed spin structure of the motif varies, such that the exact mapping from physical space to order parameter space changes within the same homotopy class, the corresponding shapes of the CD and PAM signals only undergo a linear shift, while the periodicities do not change (see Supplementary Note 1). Therefore, the topological robustness is also reflected in this type of X-ray scattering measurement.

### Experimental demonstration

**Figure 3:**
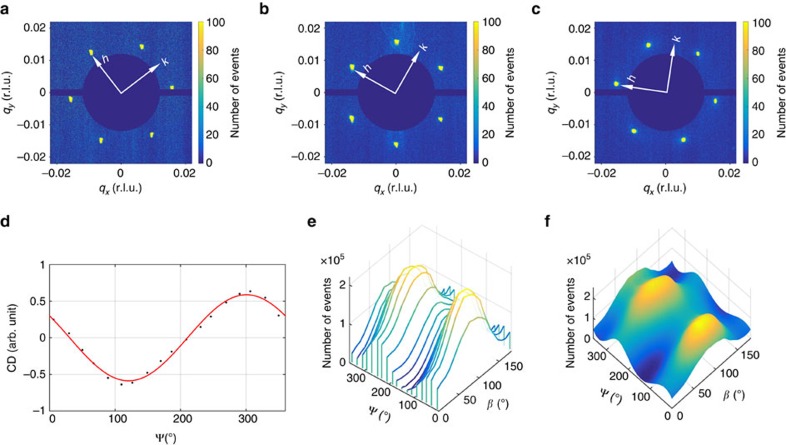
Experimental determination of the topological winding number. (**a**–**c**) Resonant soft X-ray magnetic diffraction from Cu_2_OSeO_3_ for three different azimuthal angles: Ψ=5.95°, 29.56° and 50.17°, respectively. The photon energy is tuned to 931.25 eV and the magnetic satellites are observed around the (001) structural peak. The figures show the reciprocal space maps within the (*hk*1)-plane, obtained by integrating the scattering intensities for left- and right-circularly polarized incident light. The coordinate system is defined in Fig. 1d. The temperature was 57 K and the applied magnetic field of 32 mT along the *z* direction, which is also parallel to the [001] crystallographic direction. (**d**) CD signal as a function of Ψ. The black dots are the measured data, while the red line is a fit using equation (3). (**e**) Measured PAM, and (**f**) interpolation obtained by fitting equation (4). The interpolated PAM shows excellent agreement with the calculated result shown in Fig. 2i, in which two humps of equal height can be observed at *β*≈90°.

To demonstrate the measurement principle, we performed experiments on single-crystalline Cu_2_OSeO_3_ with the setup sketched in Fig. 1d. At 57 K, and in an applied magnetic field of 32 mT, the skyrmion lattice phase emerges, manifesting itself as a hexagonal lattice of *N*=1 topological motifs. The lattice gives rise to the six-fold-symmetric diffraction pattern in reciprocal space, shown in Fig. 3a. The six sharp first-order magnetic peaks correspond to the ‘unit cell' of the skyrmion lattice, with one of them locked along *h*, that is, the [100] crystallographic direction in real space. This is due to the higher-order magnetic anisotropy of the material^34^,^36^,^37^. Note that the coordinates (*q*_*x*_, *q*_*y*_) used here, as defined in Fig. 1d, are independent of the crystallographic directions. Therefore, by rotating Ψ, the same modulation wavevectors rotate accordingly in the coordinate system (see Fig. 3b,c). The measured CD intensity as a function of Ψ (see Fig. 3d) shows exactly one period when the X-rays map the physical space once, suggesting that the skyrmion motif has a winding number of *N*=1. Moreover, as shown in Fig. 3e,f, the PAM is in excellent agreement with the theoretical calculations presented in Fig. 2i. The equal height of the two humps confirms the *N*=1 topology of this material.

## Discussion

**Figure 4:**
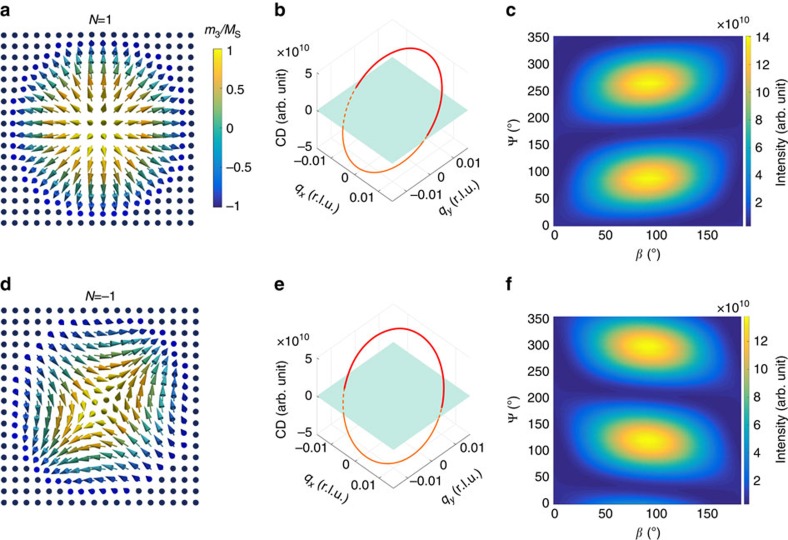
Robustness of the measurement principle. (**a**) Spin configuration of a Néel-type skyrmion with *N*=1, (**b**) calculated CD profile, and, (**c**) calculated PAM. (**d**) Spin configuration of an anti-skyrmion with *N*=−1. Note that the anti-skyrmion, as well as other topological entities with negative topological numbers, are not energetically stable states. Nevertheless, our experimental principle can be applied, as shown in **e**,**f** in which the CD profile and PAM periodicity suggest the correct absolute value of the winding number. Moreover, negative winding numbers give rise to a fundamentally different PAM shape of the humps. This provides an additional way to distinguish positive from negative values of *N*.

Another type of *N*=1 system, which does not carry chirality, is the so-called Néel-type skyrmion^33^ (see Fig. 4a). Its spin texture has a different appearance; however, it is topologically equivalent to the other skyrmion form. Consequently, the CD profile in Fig. 4b shows the same periodicity; however, a constant phase shift, as compared with Fig. 2e, appears. The phase shift, on the other hand, is due to the different mapping of the spin configuration under a continuous transformation. The analytical solution takes the value of Φ_1_=*π*/2 for equation (4). The same behaviour is found for the PAM, as shown in Fig. 4c, for which the shape and height of the two humps are essentially the same as in Fig. 2i; however, the entire pattern undergoes a linear shift along Ψ. The analytical solution takes the value of Φ_2_=*π*/6 for equation (3). Moreover, if the winding number is negative (see Fig. 4d), the CD signal still shows the same periodicity, that is, it does not distinguish between *N* and −*N*. However, the appearance of the humps is fundamentally different (compare Fig. 4f with Fig. 4c).

In summary, we have demonstrated that for a long-wavelength magnetically ordered system, the topological winding number of the motif can be unambiguously determined by polarized X-rays. First, our polarization-dependent REXS strategy is a direct measurement method as the winding number is naturally encoded in the underlying physics of the light–matter interaction, and is explicit in the measurement principle, expressed in equation (3) and (4). Second, although we used resonant soft X-ray diffraction for the demonstration of the measurement principle, the fundamentally same theory, with slight modifications, can be applied to the hard X-ray wavelength regime as well. It can further be expanded to non-resonant magnetic X-ray scattering by adding certain corrections. Third, this experimental technique can be applied to a wide range of magnetic systems, including both metallic and insulating materials, as well as other genres of materials that host topologically ordered spin systems, making it a general experimental principle.

## Methods

### Polarization-dependent resonant magnetic X-ray scattering

For the derivation of the polarization-dependent REXS signal of an *N*-skyrmion system, we start from the basic resonant X-ray scattering process from chiral magnets^39^,^40^,^41^,^42^. For a single magnetic ion at site *n* carrying a moment **m**_*n*_ the resonant scattering form factor in the electric-dipole approximation takes the form:


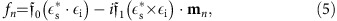


where 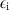 and 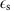 are the polarization unit vectors of the incident and outgoing X-rays, and the asterisks denotes the complex conjugate. The 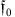 and 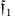 terms are the charge and the linear magnetic part of the energy-dependent resonance amplitude, respectively. The 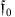 term describes the anomalous charge scattering at resonance, which is added to the Thomson scattering part. The 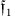 term describes resonant magnetic scattering, which can be of the same order of magnitude as charge scattering. In equation (5), we have neglected the term that is quadratic in the magnetization as it is much smaller than the leading terms, and which gives rise to higher-order effects.

In the first Born approximation, the diffraction intensity for the scattering vector **Q**=**k**_s_−**k**_i_ from a periodic lattice with sites **r**_*n*_ can be written as


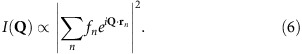


The complex amplitudes 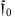 and 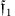 are energy-dependent. Here we take them as constant, since in our numerical calculations the photon energy is not varied.

The coordinate system used to carry out the polarization-dependent study of the scattering cross-section is shown in Fig. 1b. The Cartesian coordinates are determined by the scattering plane (containing **Q**), that is, the *x*–*z-*plane in this case. The *y* axis is perpendicular to this plane. This defines the three components for the magnetization vectors, as well as the reciprocal space coordinates (*q*_*x*_, *q*_*y*_, *q*_*z*_). Thus, **k**_i_=*k*(cos *α*, 0, sin *α*), **k**_s_=k(cos *α*, 0, −sin *α*), **k**_s_ × **k**_i_=*k*(0, −2 cos *α* sin *α*, 0), where the magnitude of the X-ray wavevector, *k*, relates to the photon energy [*k*=2*π*/*λ*=*E*/(*ħc*)].

In the Poincaré-Stokes representation, the polarization of the incident X-rays is characterized by **P**=(*P*_0_, *P*_1_, *P*_2_, *P*_3_). For left- and right-circularly polarized light, *P*_0_=1, *P*_1_=*P*_2_=0, *P*_3_=±1. For linearly polarized light, *P*_0_=1, *P*_1_=cos 2*β*, *P*_2_=sin 2*β*, *P*_3_=0.

For a magnetic system carrying incommensurate magnetic modulations, magnetic diffraction occurs as satellites **q** surrounding the structure peak **G**, so that **Q**=**G**+**q**. Therefore, the charge and magnetic part of the diffraction can be separated, and no charge-magnetic interference term is expected. Consequently, at the diffraction condition for the periodic magnetic structure, the scattering intensity is described by the magnetic part (that is, second term of equation (5)). It is straightforward, but rather tedious, to derive the intensity for the magnetic scattering, which is given by^41^:


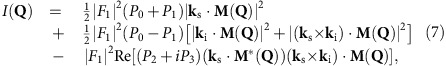


where *F*_1_ is the energy-dependent resonant term, and **M**(**Q**) is the Fourier transform of the real-space magnetic moment modulation **m**(**r**) at **Q**. Note that **k**_i_ and **k**_s_ are tuned to fulfil the diffraction condition for **Q**.

### X-ray polarization dependence of the winding number

As shown in Fig. 1a, a one-dimensional proper-screw helix pitch^43^, otherwise called ‘Bloch-type' helix, propagating along *x*, can be written as


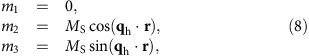


where **q**_h_ is the helix propagation wavevector. We define a base position of the helix such that **q**_h_ is along the *x* axis, this also corresponds to Ψ=0°. While the X-rays probe the physical space at an finite angle Ψ, the order parameter space magnetization profile rotates the base helix position specified by equation (8) by *N*Ψ within the *q*_*x*_–*q*_*y*_-plane. By applying the rotation matrix 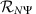
=
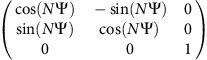
 to equation (8), the rotated magnetic structure becomes


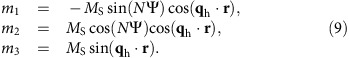


To meet the diffraction condition for **Q**=**G**+**q**_h_ at Ψ, one has to bring **Q** into the scattering plane. In a common four-circle diffractometer, this is achieved by compensating the diffraction offset with the other two rotation axes, that is, the *α* axis, and the *κ* axis, which is perpendicular to both the *α* and Ψ axes. As a consequence, the components of the magnetic structure transform into:


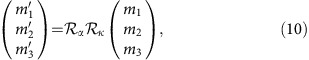


where 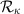 and 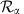 are the rotation matrices corresponding to the *κ* and *α* rotation axes, and the combination of the two rotations brings **Q** into the scattering plane for the diffraction condition.

However, it is essential to note that this change would be negligible for most of the long-wavelength modulated magnetic structures. For example, Cu_2_OSeO_3_ has *qa*=0.0158 (ref. 37), where *a* is the lattice constant. Therefore, for **G**=(0, 0, 1), **Q**=**G**+**q**_h_, the change of *α* is less than 0.9° for all Ψ angles. This makes 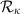 and 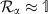. Therefore, the long-wavelength approximation suggests 
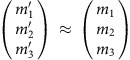
, as well as **M**(**Q**)≈**M**(**q**_h_), and we can take one angle *α* for the diffraction condition of all Ψ positions.

Thus, the Fourier transform of equation (9) at the diffraction condition of **q**_h_ takes the form


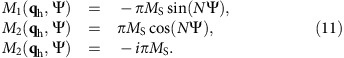


Inserting equation (11) into equation (7), and evaluating the expressions described above, the CD profile is obtained as


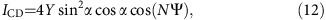


where 
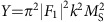
. However, note that the CD intensity is zero for even values of *N*. This condition is not captured by the analytical relationship of equation (12); however, it can be generalized from the numerical calculations. The reason why the CD vanishes is discussed in the main text, and is based on the assumption that, for an example, an *N*=2 skyrmion can be considered as two *N*=1 skyrmions pulled together^2^. The ‘chirality cancelling' effect does not occur for odd winding number motifs; however, it exists for all even winding numbers. Moreover, as will be discussed shortly, adding another phase factor Φ_1_ is also necessary for generalizing the CD relationship to *N*-skyrmions.

The linear polarization dependence can be derived as


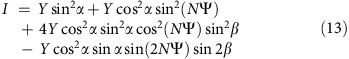


Equations (12) and (13) are the foundation of our measurement principle, and are derived based on a standard one-dimensional helix structure. Therefore, this analytical form is only valid for ‘standard' types of spin configurations, for an example, *N*=1 chiral skyrmions with *χ*=±*π*/2. However, in principle, there is an infinite number of homotopies for a certain winding number, that is, the same topological property will always hold if continuous transformations are acting on a ‘standard' skyrmion configuration, as we have used and derived so far. For example, if the one-dimensional helix takes other forms, such as a cycloidal type structure^33^, the overall spin texture will change while the winding number remains invariant. As shown in the Supplementary Note 1, this degree of freedom is dealt with by adding a phase factor to equations (12) and (13), which makes the measurement principle generally valid for all cases.

### Numerical calculations

Numerical calculations were carried out using the materials parameters of Cu_2_OSeO_3_, that is, a helix pitch of 60 nm. This leads to a skyrmion core-to-core distance of ∼69.28 nm, as well as a wavevector of ∼0.015 r.l.u. Resonant X-ray scattering at the Cu *L*_3_ edge with a photon energy of 931.25 eV gives *k*=2*π* × 4.7187, nm^−1^, with *α*≈48.24° for the (0, 0, 1) diffraction peak. In the calculation, *F*_1_ and *M*_S_ are kept constant as the CD profile and PAM are measured for the same photon energy and temperature. For detailed numerical results, please see Supplementary Note 1.

### REXS

Resonant soft X-ray scattering experiments were carried out in the RASOR diffractometer on beamline I10 at the Diamond Light Source (UK). Single crystals of Cu_2_OSeO_3_ are pre-characterized by X-ray diffraction and electron back-scattering diffraction to confirm the crystalline quality and single chirality. Magnetometry measurements were performed to map out the magnetic phase diagram. The polished crystal surface was (001)-oriented for the subsequent resonant X-ray scattering measurements.

The incident soft X-ray beam with variable polarization was tuned to the Cu *L*_3_ edge. The experimental geometry is shown in Fig. 1d. The scattered beam is captured by either a CCD camera or a photodiode point detector. The modulated magnetic structure leads to satellites surrounding the structural peaks in reciprocal space. Further details about the experimental methods on resonant soft X-ray scattering can be found in refs 37, 42. Polarization-dependent measurements are performed by varying the incident light polarization, while measuring the scattering intensities for different diffraction conditions for varying Ψ.

### Data availability

The data that support the findings of this study are available from the corresponding author on request.

## Additional information

**How to cite this article:** Zhang, S. L. *et al*. Direct experimental determination of the topological winding number of skyrmions in Cu_2_OSeO_3_. *Nat. Commun.* **8,** 14619 doi: 10.1038/ncomms14619 (2017).

**Publisher's note**: Springer Nature remains neutral with regard to jurisdictional claims in published maps and institutional affiliations.

## Supplementary Material

Supplementary Information Supplementary Figures, Supplementary Notes and Supplementary References

Supplementary Movie 1 Covering N=1.mov. Demonstration of the topological covering concept based on one-dimensional helix chains for N = 1, 2, 3 and 6 skyrmions. The left panel shows the order-parameter-space coverage and the right panel the corresponding magnetisation configuration.

Supplementary Movie 2 Covering N=2.mov. Demonstration of the topological covering concept based on one-dimensional helix chains for N = 1, 2, 3 and 6 skyrmions. The left panel shows the order-parameter-space coverage and the right panel the corresponding magnetisation configuration.

Supplementary Movie 3 Covering N=3.mov. Demonstration of the topological covering concept based on one-dimensional helix chains for N = 1, 2, 3 and 6 skyrmions. The left panel shows the order-parameter-space coverage and the right panel the corresponding magnetisation configuration.

Supplementary Movie 4 Covering N=6.mov. Demonstration of the topological covering concept based on one-dimensional helix chains for N = 1, 2, 3 and 6 skyrmions. The left panel shows the order-parameter-space coverage and the right panel the corresponding magnetisation configuration.
