# Supplementary material for: Direct experimental determination of the topological winding number of skyrmions in Cu2OSeO3
Source: Nat Commun. 2017 Feb 24;8:14619. doi: 10.1038/ncomms14619 (PMC5333093; doi:10.1038/ncomms14619)
Supplement: Supplementary Information — Supplementary Figures, Supplementary Notes and Supplementary References [file ncomms14619-s1.pdf]

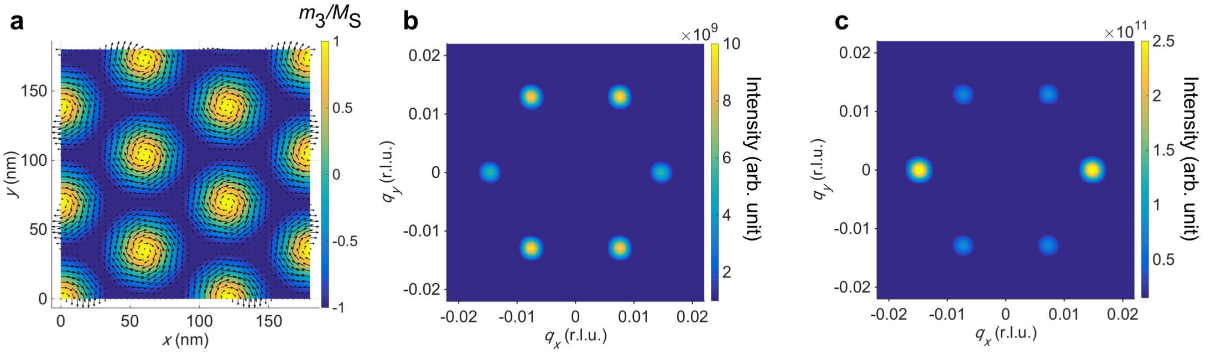

Supplementary Figure 1. **Magnetisation configuration used for REXS simulations.** **a**, Periodic real-space lattice of the topological motif. The cropped image shows a hexagonally ordered array of $N = 1$ motifs, i.e., the skyrmion lattice as observed in Cu_2OSeO_3. Note that, in principle, the motif ordering can take any symmetry, such as two-fold, three-fold, or four-fold, which always give rise to the same polarisation-dependent REXS results. Calculated magnetic diffraction patterns in reciprocal space for the real-space spin configuration shown in **a**, for **b** σ-polarised and **c** π-polarised incident x-rays, respectively. The material parameters used in the calculation are those of Cu_2OSeO_3 and the photon energy is 931.25 eV.

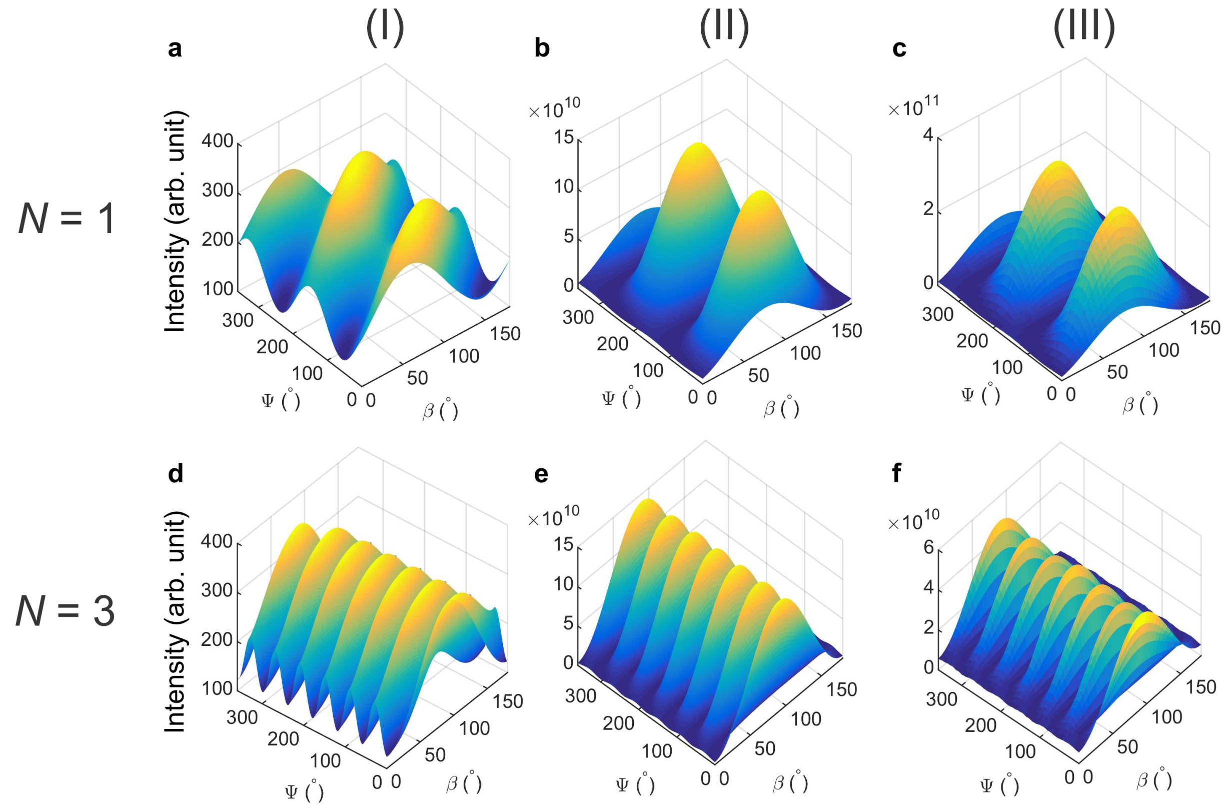

Supplementary Figure 2. **Comparison between different simulation methods.** **a-c**, Polarisation-azimuthal maps for the $N = 1$ motif and, **d-f**, for the $N = 3$ motif, calculated using three different methods: **a,d** are obtained directly using analytical solutions, corresponding to Method (I) (see text for details); **b,e** are calculated numerically using Method (II); and **c,f** are rigorous numerical calculations using Method (III).

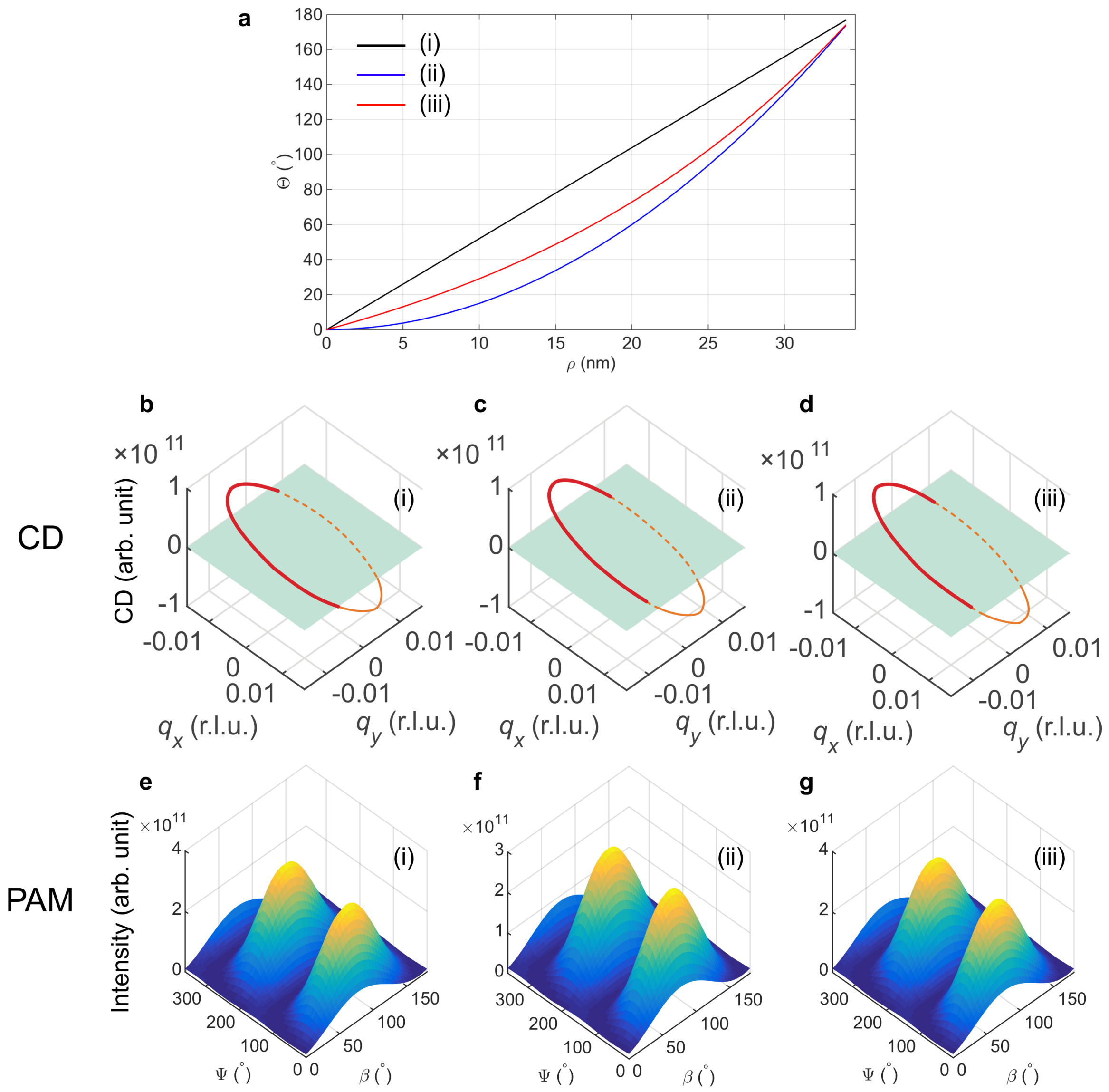

Supplementary Figure 3. **Robustness of the measurement principle for varying radial profile. a**, Three different $\Theta(\rho)$ profiles that govern different radial spin distributions, labelled as (i), (ii), and (iii), are used for the subsequent numerical calculations. Note that profile (i) represents a linear relationship, which is equivalent to the one-dimensional helix modulation case. **b-d**, Circular dichroism (CD) profiles, and, **e-g**, polarisation-azimuthal maps (PAMs) calculated based on the three different radial functions. It can be seen that both CD and PAM are independent of the radial profile, confirming the robustness of the measurement principle.

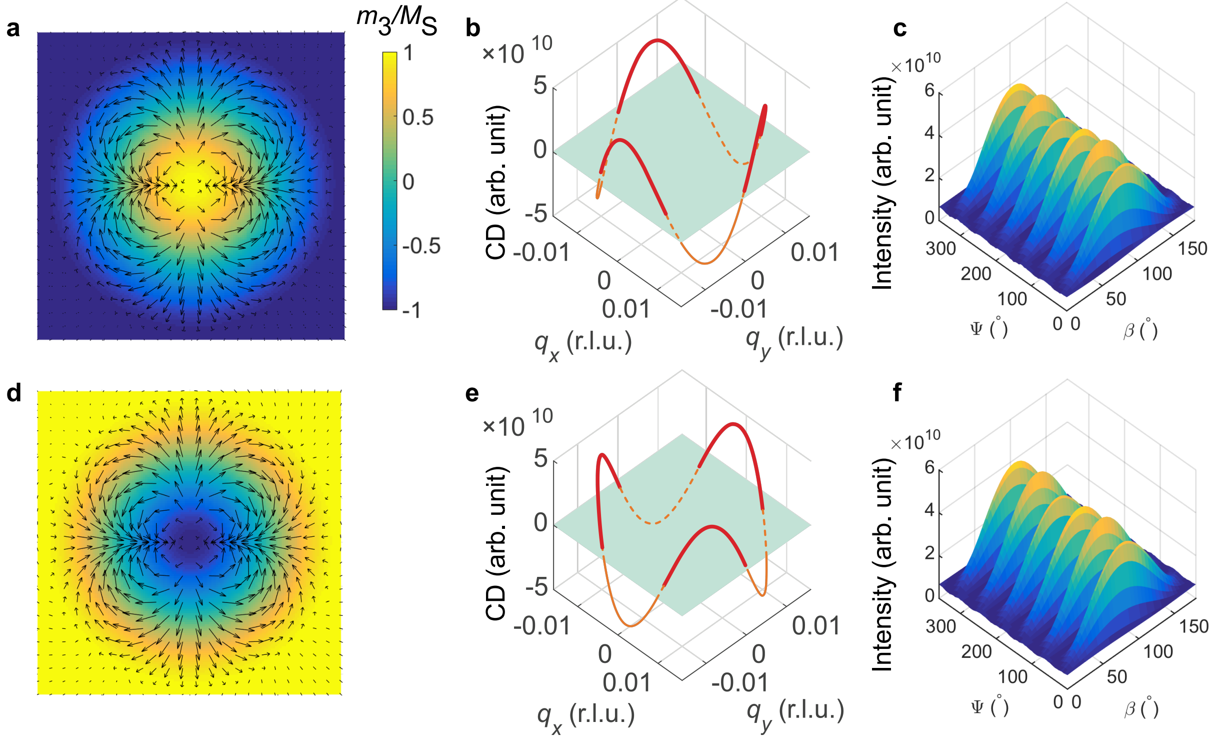

Supplementary Figure 4. **Robustness of the measurement principle for varying χ and λ.** **a**, Real-space spin configuration with $N = 3$, however, having a different χ value compared to the configuration shown in Fig. 2c in the main text. **b**, Calculated circular dichroism (CD) profile, and **c**, polarisation-azimuthal map (PAM). **d**, Another $N = 3$ spin configuration with opposite polarity λ compared to **a**. **e**, Calculated CD profile, and **f**, PAM pattern. Note that the periodicities of the intensity modulation for both CD and PAM are independent of χ and λ, again confirming the robustness of the measurement principle.

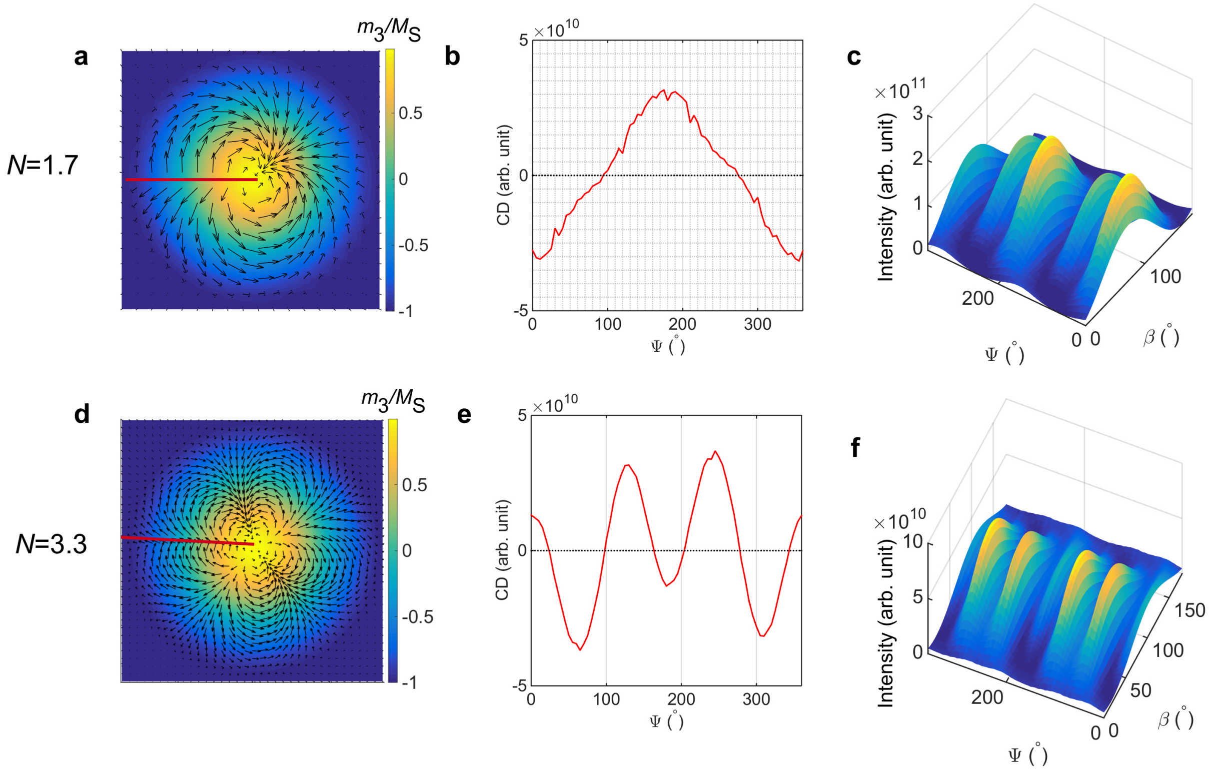

Supplementary Figure 5. **Simulation results for non-integer winding numbers. a,d**, Real-space spin configuration, and **b,e**, Circular dichroism, and, **c,f**, polarisation-azimuthal maps for, **a-c**, $N = 1.7$ and, **d-f**, $N = 3.3$, respectively.

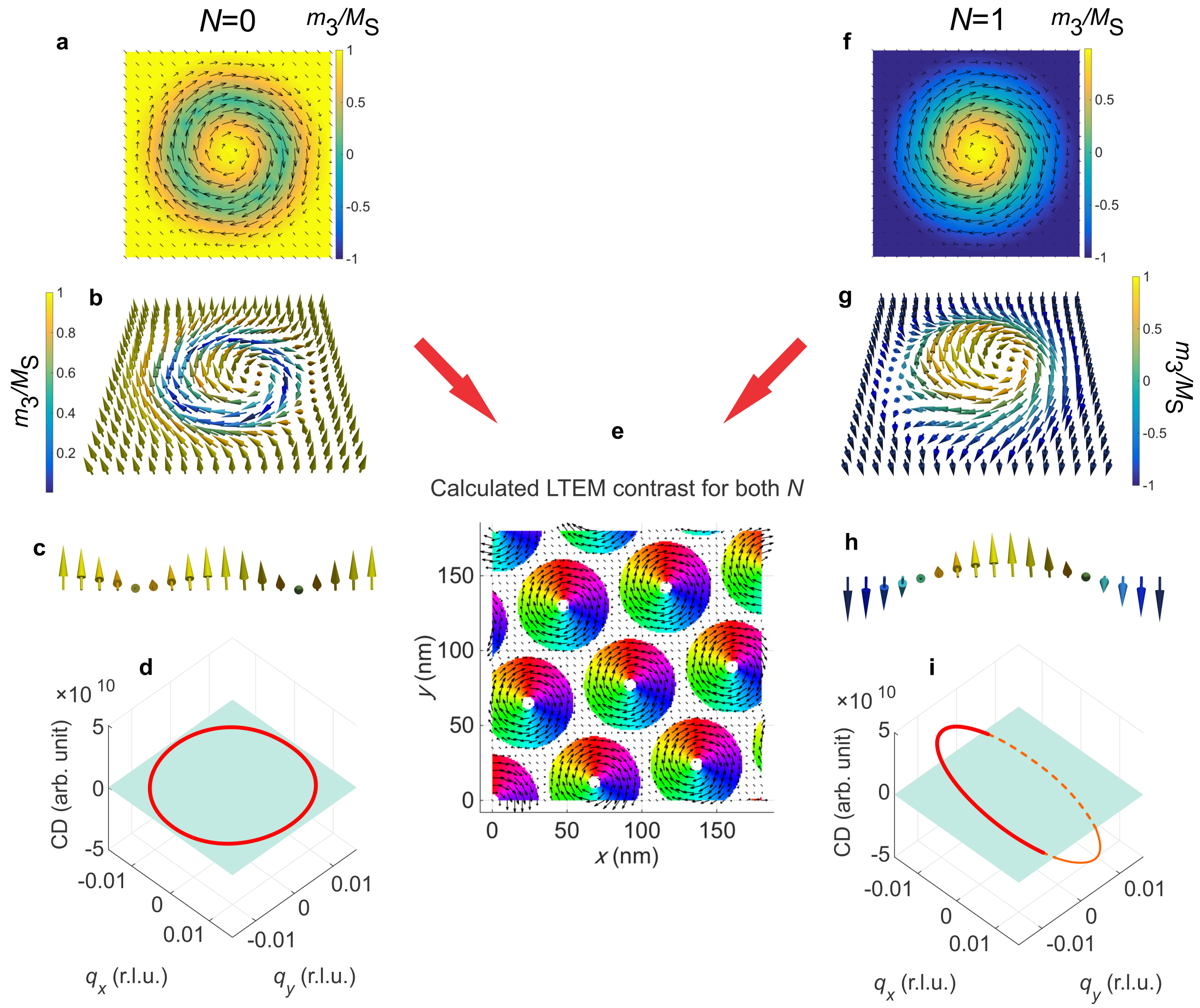

Supplementary Figure 6. **Determination of the winding number using LTEM and REXS. a,b**, Magnetisation configuration for a topologically trivial $N = 0$ vortex, and, **c**, underlying one-dimensional helix. **e**, Simulated LTEM (Lorentz transmission electron microscopy) image for both the $N = 0$ and $N = 1$ cases, in which essentially only the m_1 and m_2 components of the magnetisation distribution are mapped using the transport-of-intensity equation method [1]. **d**, Circular dichroism (CD) plot showing a fully suppressed signal for the $N = 0$ case. **f,g**, Magnetisation configuration for a (standard) $N = 1$ chiral skyrmion, and, **h**, underlying one-dimensional helix. Whereas LTEM gives the same contrast for $N = 0$ and $N = 1$, the CD plot in **i** shows the contrast consistent with an $N = 1$ skyrmion.

Supplementary Note 1. ROBUSTNESS OF THE MEASUREMENT PRINCIPLE

Supplementary Figure 1a shows as an example of the cropped simulation object with $N = 1$. The motifs are generated based on Eq. (2) (see main text), and assembled into a hexagonally ordered two-dimensional periodic lattice. The symmetry of the lattice does not change any results, as we only concentrate on one wavevector at different Ψ. In the calculations we use a periodic motif lattice size of 300×300 nm^2. The calculated diffraction intensity, represented in reciprocal space using Eq. (7) (see main text), is plotted in Supplementary Figure 1b and 1c, where the incoming light is at $\beta = 0°$ and $90°$, respectively. On the other hand, we use the one-dimensional helix approximation method to perform the numerical calculations for the same object, in order to confirm the equivalence of both methods.

In summary, three theoretical methods are used for calculating the CD and PAM as a function of the topological winding number, in order to demonstrate the consistency of the results:

(I) Analytical solution based on Eq. (3) for PAM and Eq. (4) for CD in the main text.

(II) Construction of the skyrmion configuration using the one-dimensional helix approximation model. For the azimuthal angle Ψ, the diffractive x-rays are sensitive to the structure factor of the spin helix, obtained by rotating the helix $N\Psi$ from the base position. The CD and PAM are subsequently calculated numerically.

(III) Generation of a two-dimensional skyrmion lattice using the rigorous solution given by Eq. (2) in the main text. The CD and PAM are then numerically obtained using Eq. (7) (see main text).

We first demonstrate the consistency between the three calculation methods, which further confirms the validity of the analytical solution. Supplementary Figure 2 shows the comparison for PAMs obtained using the different methods. The top and bottom rows correspond to $N = 1$ and $N = 3$ systems, respectively. Plots in the left column arise directly

from Eq. (4) (see main text) [method (I)]. The plots in the middle column are showing the numerical calculation results obtained using method (II), while those in the right column are the rigorous numerical results using method (III). As can be seen, the general multiple hump features are consistent, which are directly linked to the winding number. The differences in some fine details are due to the exact values of the parameters used in the numerical calculations, such as M_S, k, and α. Also, we found that the real-space boundaries, and the simulation mesh structure can cause slight deviations compared with Eq. (4) (see main text), which can thus be regarded as simulation artefacts. This consistency demonstrates that the concept of the one-dimensional spin helix approximation indeed allows us to construct a topological spin structure with winding number N, and that the analytical solution derived this way is valid.

Next, we show that other parameters that describe a general N-skyrmion, i.e., the $\Theta(\rho)$ function, λ, and χ, do not affect the validity of the measurement principle. First, we discuss the dependence on $\Theta(\rho)$, which can be regarded as the radial profile of the out-of-plane magnetisation component, m_3, from the core to the boundary. For an axially symmetric entity, $\Theta(\rho)$ usually satisfies the Euler equation [2]. However, for our purpose, changing this profile does not affect the CD and PAM features at all, as long as the boundary condition is met. We use the $N = 1$ skyrmion as an example. As shown in Supplementary Figure 3a, $\rho = 0$ nm corresponds to the real-space skyrmion vortex core, while the maximum value of ρ in the plot corresponds to the radius of a skyrmion disk in our calculations. Its value is related to the helix pitch by $(60 \text{ nm} \times 2/\sqrt{3})/2$. Three different $\Theta(\rho)$ profiles, labelled as (i), (ii), and (iii), are used in the simulation for the purpose of comparison. All of them have the same homotopy. As shown in Supplementary Figures 3b-g for both CD and PAM, the three different profiles do not induce any significant differences. They are nearly identical, except for the slight difference in amplitude of the cross-section. This is to be expected as the measurement principle specifies the measurement under diffraction conditions, therefore the detailed modulation profile along the propagation direction is only reflected in the total scattering amplitude, not in the polarisation dependence.

Second, we discuss the influence of χ and λ on the CD and PAM patterns. Supplementary Figure 4a shows another $N = 3$ topological object, which is essentially a continuous transformation from the object shown in Fig. 2c (see main text). This homotopic transformation can be achieved by adjusting χ. As shown in Supplementary Figures 4b and 4c,

compared to Fig. 2g and 2k in the main text, the CD and PAM patterns have identical periodicities, and the only difference is a linear phase shift. This is valid for all cases in our numerical studies. Moreover, as shown in Supplementary Figure 4d-f, flipping the polarity of the topological object does not alter the PAM, however, it imposes a phase shift on the CD profile. Therefore, the use of the phase parameters Φ_1 and Φ_2 in Eqs. (3) and (4) (see main text) can generalise the principle to all homotopies arising from variations in χ and λ.

To briefly summarise, our polarisation-dependent REXS method, represented by the circular dichroism plots and the polarisation-azimuthal maps, is only sensitive to the winding number and has a one-to-one correspondence to this topological quantity. Any homotopy change will not affect the outcome of the measurement. In other words, the method itself can be seen as 'topologically protected'.

Supplementary Note 2. NON-INTEGER WINDING NUMBERS

Here we discuss the case of non-integer winding numbers. Note that non-integer winding numbers correspond to energetically unstable states, due to the appearance of the singularities within their spin structures. As shown in Supplementary Figures 5a and 5d, the abrupt change of the spins across the red lines will cost extremely high energy, leading to the unstable states. However, we will calculate the corresponding CD and PAM in order to demonstrate that our new technique is only sensitive to spin configurations with integer topological winding numbers. Supplementary Figures 5a-c show the magnetisation distribution, CD and PAM for a $N = 1.7$ motif lattice. First, the CD shape is largely distorted from a well-defined sinusoidal curve shape. Second, in PAM, the humps are no longer of equal height due to the non-integer topology. These features can also be found for the $N = 3.3$ case, shown in Supplementary Figures 5d-f. The asymmetry is even more pronounced in CD, in which the periodically modulated peaks do not have equal height. This is also clearly shown in their PAM relationship.

Supplementary Note 3. DETERMINATION OF THE TOPOLOGICAL WINDING NUMBER USING LORENTZ TRANSMISSION ELECTRON MICROSCOPY

Finally, we show an example of an attempt to obtain the topological winding number from Lorentz transmission electron microscopy (LTEM) images. Due to the measurement principle of LTEM [1], the out-of-plane magnetisation component (m_3) is not obtained. Nevertheless, as described by Eq. (1) in the main text, the m_3 component is a necessary parameter that also governs the winding number. As a result, the LTEM image, as shown in Supplementary Figure 6e, will appear identical for certain $N = 0$ vortices and $N = 1$ skyrmions. Consequently, LTEM can not be used for the unambiguous determination of the winding number as many different spin configurations can essentially give the same LTEM contrast.

On the other hand, as expressed by Eq. (7) (see main text), polarisation-dependent REXS is sensitive to all three magnetisation components. As a consequence, the calculated CD signal as shown in Supplementary Figure 6d will be suppressed for $N = 0$ type of vortices, while PAM shows two humps. Combining the CD and PAM results, one can unambiguously conclude that the motif is a $N = 0$ vortex. This is in stark contrast to the CD and PAM results for an $N = 1$ skyrmion, as shown in Supplementary Figure 6i. Therefore, our method is a direct experimental technique that can accurately measure N.

Supplementary References

[1] X. Z. Yu, Y. Onose, N. Kanazawa, J. H. Park, J. H. Han, Y. Matsui, N. Nagaosa, and Y. Tokura, "Real-space observation of a two-dimensional skyrmion crystal," Nature **465**, 901–904 (2010).

[2] S. Zhang, A. A. Baker, S. Komineas, and T. Hesjedal, "Topological computation based on direct magnetic logic communication," Sci. Rep. **5**, 15773 (2015).
